# Computational Fluid Dynamics-Driven Comparison of Endovascular Treatment Strategies for Penetrating Aortic Ulcer

**DOI:** 10.3390/jcm14041290

**Published:** 2025-02-15

**Authors:** Katia Capellini, Emanuele Gasparotti, Vincenzo Castiglione, Cataldo Palmieri, Sergio Berti, Antonio Rizza, Simona Celi

**Affiliations:** 1 BioCardioLab, U.O.C. Bioingegneria, Fondazione Toscana Gabriele Monasterio, 54100 Massa, Italy; kcapellini@ftgm.it (K.C.); gasparotti@ftgm.it (E.G.); 2 U.O.C. Cardiologia e Medicina Cardiovascolare, Fondazione Toscana Gabriele Monasterio, 56124 Pisa, Italy; vcastiglione@ftgm.it; 3 Health Science Interdisciplinary Center, Scuola Superiore Sant’Anna, 56127 Pisa, Italy; 4 U.O.C. Cardiologia Diagnostica e Interventistica, Fondazione Toscana Gabriele Monasterio, 54100 Massa, Italy; palmieri@ftgm.it (C.P.); berti@ftgm.it (S.B.); rizza@ftgm.it (A.R.)

**Keywords:** penetrating aortic ulcer, acute aortic syndrome, aortic endograft, computational fluid dynamics

## Abstract

**Background:** Penetrating aortic ulcer (PAU) is an acute aortic syndrome characterized by a high rupture risk. There are several PAU-treatment procedures indicated for the management of this pathology associated with different effects on vessel morphology and hemodynamics. A deep evaluation of the different types of treatment may be helpful in decision making. Computational Fluid Dynamics (CFD) is a powerful tool for detailed inspection of cardiovascular diseases. The aim of this work was to implement a comparative analysis based on CFD evaluation of the effects of two type of PAU treatments. **Methods:** Thoracic endovascular aortic repair (TEVAR) with a left subclavian artery (LSA) branched aortic endograft (SBSG) and a hybrid approach including TEVAR and carotid-LSA bypass were considered. Aortic anatomical models were created from computed tomography (CT) images acquired before and after PAU treatment with SBSG for three patients. Starting from these models, a new aortic geometry corresponding to the outcome of the hybrid strategy was generated. Morphological analysis and CFD simulations were carried out for all aortic models to evaluate LSA outflow for the same predefined boundary conditions. **Results:** Reductions in LSA diameter were found between aortic models before and after the SBSG (18.2%, 20.8%, and 12.4% for CASE 1, CASE 2, and CASE 3, respectively). The flow rate at LSA changed between pre-configuration and aortic configuration after the PAU treatments: an averaged decrement of 1.08% and 7.5% was found for SBSG and the hybrid approach, respectively. The larger increase in pressure drop between the aortic arch and the LSA extremity was shown in the hybrid approach for all cases. **Conclusions:** CFD simulations suggest that SBSG preserves LSA perfusion more than a hybrid strategy and has less impact on thoracic aorta hemodynamics.

## 1. Introduction

Penetrating aortic ulcer (PAU) is a severe form of acute aortic syndrome (AAS) that presents challenges in clinical management due to its high risk of rupture, with rates up to 40% [1]. Characterized by an ulceration of the aortic wall that penetrates through the internal elastic lamina and extends into the media, PAU predominantly affects older individuals, especially those with a history of atherosclerosis, hypertension, and smoking [1,2,3]. Given the complex nature of the condition, careful consideration of treatment options is essential, including surgical, hybrid, and thoracic endovascular aortic repair (TEVAR), which involves placing a covered stent.

Current guidelines from Europe and the United States advise considering surgical intervention for lesions in the ascending aorta. However, due to the risks associated with open surgery, particularly in older patients with significant comorbidities, endovascular approaches have become increasingly attractive. These procedures, especially with the advent of customizable and branched prostheses that protect the epiaortic vessels, have gained prominence for their minimally invasive nature and ability to reduce perioperative risks in high-risk patients [4,5]. In particular, left subclavian artery (LSA) revascularization may be necessary during the TEVAR procedure due to the eventual coverage of the artery origin by the endograft. Different methods are adopted to perform the revascularization and their clinical efficacy, advantages, and disadvantages are explored in the literature [6]. Chimney stent (CS) and in situ fenestration (ISF) techniques involve the placement of additional stent grafts. In particular, the CS approach consists in the deployment of a stent in the LSA branch parallel to the endograft inserted in the aorta via the TEVAR procedure [7,8], while the ISF technique involves penetrating the previously placed aortic endograft with TEVAR through various strategies such as the tip of a guide wire or the use of a laser and then implanting a further stent into the obtained fenestration with its extremity within the aortic endograft [9,10]. Among the LSA revascularization techniques, the surgical placement of a short prosthetic conduit between left common carotid artery (LCCA) and LSA (LCCA-LSA bypass) is the most diffused method [11]. The combined use of this technique and TEVAR is a hybrid approach with the related surgical risks [12].

The adoption of a single-branched stent graft (SBSG) is instead a total endovascular strategy developed in recent years [4,13] which is associated with a low risk of endoleak complication [14]. Given the changes in vessel geometry due to interventions and the influence of geometry on fluid dynamic patterns [15], the effects of PAU-treatment options on aorta and supra-aortic branch hemodynamics should be investigated for an accurate evaluation of these strategies. Numerical simulations are widely diffused in cardiovascular research to investigate biomechanics and hemodynamics of several districts [16,17,18,19]. Computational fluid dynamics (CFD) and fluid structure interaction (FSI) simulations have been successfully adopted to assess the blood patterns of the thoracic aorta (TA), including supra-aortic vessels [20,21,22,23,24], mainly thanks to their ability to compute clinically relevant hemodynamic indices with high accuracy, overcoming the dependence on spatial resolution affecting their in vivo estimation [25,26]. The vessel fluid dynamics has been investigated for TEVAR strategies implementing LSA revascularization allowing for detailed analysis of blood flow patterns and pressure changes within the aorta, offering insights into the effectiveness of various interventions [5,27,28,29,30]. In our previous work, CFD simulations were used to assess the LSA flow rate after a endovascular PAU treatment in order to preserve the patency of a coronary artery bypass graft [5].

The aim of this work was to evaluate TA hemodynamics using CFD simulations on computed tomography (CT) images before and after PAU endovascular treatment with an SBSG, including a component for the LSA and its relations with vessel morphology. Moreover, a CFD comparative analysis with a virtual hybrid approach with LCCA-LSA bypass was performed to clarify the hemodynamic consequences of each approach and their implications for patient outcomes.

## 2. Materials and Methods

### 2.1. Image Data

Two CT image datasets of three patients who underwent endovascular treatment for PAU pathology with a single-branched aortic stent graft (Endovastec^™^ Castor^™^) were retrospectively analyzed. The adoption of SBSG allows the preservation of LSA thanks to a branch section on the endograft. That section is kept open thanks to a steel ring embedded at the branch origin [31].

**Figure 1:**
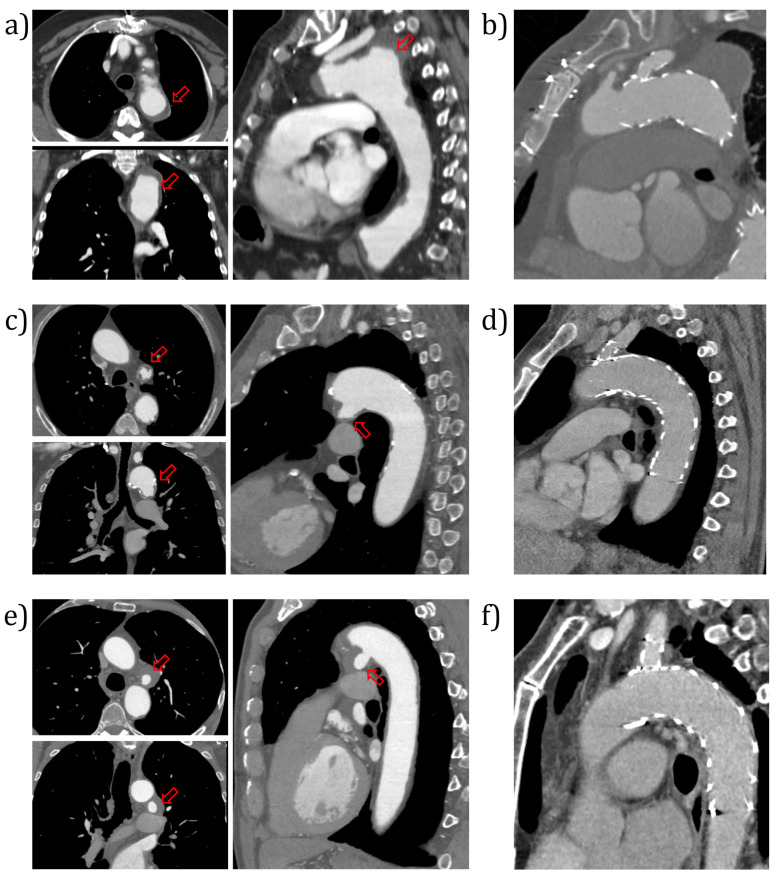
CT acquisitions pre-intervention (**a**,**c**,**e**) and CT acquisitions post-SBSG treatment (**b**,**d**,**f**) for the three cases. PAUs are indicated by red arrows.

For each patient, CT images acquired before (Figure 1a,c,e) and after the procedure (Figure 1b,d,f) were considered, the follow-up exams were conducted thirty days after the intervention to assess the proper placement of the endograft and the absence of endoleaks. The images were acquired with a 320-detector scanner (Toshiba Aquilon One, Toshiba, Japan), after the injection of a contrast medium. The datasets showed a pixel resolution value ranging between 0.468 mm to 0.873 mm and a slice thickness in the range of 0.5–2.0 mm.

### 2.2. Image Processing and Morphological Analysis

A segmentation procedure was performed on each dataset by applying a threshold algorithm to select the lumen region by using 3D Slicer 4.11.0 software [32]. Three-dimensional models of thoracic aorta including supra-aortic branches in stereolithography (STL) file format were obtained for the datasets acquired before (PRE) the intervention and at the follow-up (SBSG). A surface mesh refining phase was implemented to overcome any artefacts resulting from the endograft presence in SBSG images with a subsequent smoothing procedure to obtain models suitable for a computational environment. A third aorta geometry (HYBRID), associated with an LCCA-LSA bypass and TEVAR treatment for PAU, was generated starting from the PRE and SBSG 3D models. For each patient, PRE and SBSG geometries were firstly registered with a roto-translation by aligning the ascending tract, the brachiocephalic artery (BCA) and the LCCA. The HYBRID geometry was generated from the region of TA, the BCA and the LCCA of the SBSG, while the LSA branch that does not present a graft cannulation was selected from the PRE 3D model. The merging of the above regions and the TEVAR coverage of the LSA were performed through a computer aided design (CAD) procedure implemented in SpaceClaim (ANSYS Inc., Canosburg, PA, USA). In particular, the LSA coverage was replicated by positioning a patch, conforming with aortic curvatures at the LSA origin. The LCCA-LSA bypass was created using a curved cylinder with an 8 mm diameter, positioned approximately 2 cm distal to the left vertebral artery in the LSA and on the lateral side of the LCCA, following surgical indications based on the anatomical features of each case.

**Figure 2:**
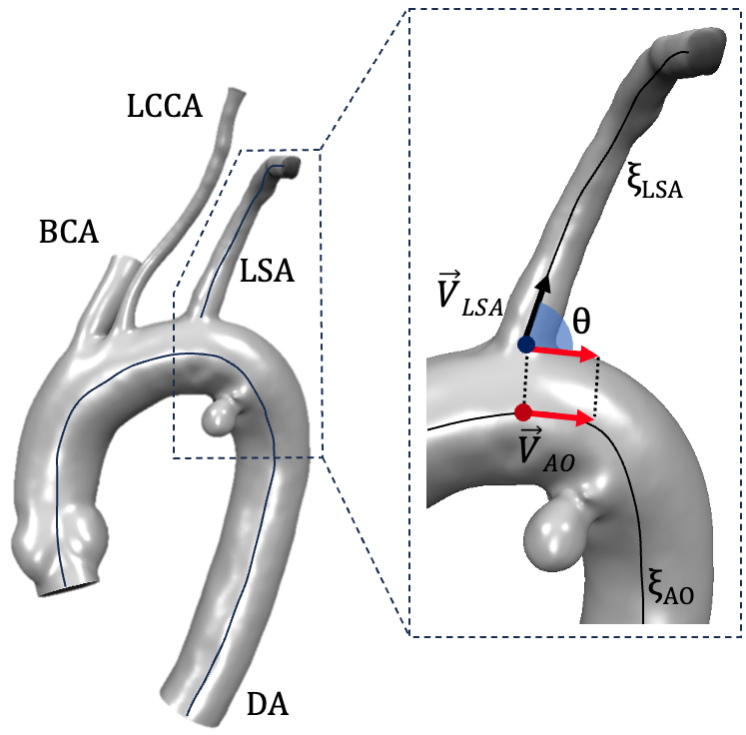
Assessment of bifurcation angle between aortic arch and LSA.

A morphological analysis of LSA was carried out for the PRE and SBSG configurations to evaluate the impact of the pre-cannuled endograft. The aorta and LSA centerlines ($\xi_{AO}$ and $\xi_{LSA}$, respectively) were computed, and cross-sections were extracted for the first segment of the LSA, starting from the LSA origin to the single branch end. For each section, the maximum diameter ($D_{max}$) was calculated together with the area. The inclination of LSA was computed by calculating the angle (θ) between the LSA vector $\left({\vec{V}}_{\;LSA}\right)$ along the $\xi_{LSA}$ and the aortic vector $\left({\vec{V}}_{\;AO}\right)$ along the $\xi_{AO}$ [33]. The ${\vec{V}}_{LSA}$ was defined between a point at the beginning of $\xi_{LSA}$ and a point at a distance equal to the LSA diameter along the $\xi_{LSA}$. The ${\vec{V}}_{\;AO}$ was defined starting from the point of $\xi_{AO}$ showing the minimum distance to the LSA origin to a point at a distance equal to the LSA diameter along the $\xi_{AO}$ toward the descending aorta (DA) (Figure 2). Moreover, the tortuosity of the centerline between the aortic inlet and the end of LSA branch was estimated. A morphological analysis was performed by a custom script implemented in the Vascular Modelling Toolkit-Python environment.

### 2.3. Computational Fluid Dynamics Simulations

CFD transient simulations were performed for the three thoracic aorta configurations (PRE, SBSG, HYBRID) for all cases. Regarding the mesh sensitivity analysis, four different decreasing mesh element sizes were considered to discretise the solid domain. The difference on the blood velocity and time averaged wall shear stress (TAWSS), with respect to each subsequent finest mesh, resulted as 0.05% and below 3%, respectively. Thus, the 3D models were discretized by a polyhedral mesh with four boundary inflation layers and an average element edge equal to 0.5 mm.

The flow dynamics behaviour was simulated by adopting the Navier–Stokes equations solved in their discrete form through the finite volume method in the Ansys^th^ Fluent^th^ (ANSYS, Inc., Canonsburg, PA, USA). Although blood is a non-Newtonian fluid, its rheological properties depend on the vessel size and for vessels much larger than the characteristic size of red blood cells, as the TA, the assumption of Newtonian behavior is widely adopted in the literature [22,34,35,36]. Hence, in this study, the blood flow was modelled as Newtonian fluid with a density of 1060 kg/m^3^ and a constant viscosity of 3.5·10^−3^ Pa·s. The artery wall was assumed rigid with no slip conditions. A representative aortic flow profile [5] was selected as the inlet boundary condition for all simulations. For the BCA, LCCA, LSA, and DA pressure outlet conditions were assumed by implementing a lumped 3-element Windkessel model (RCR Windkessel), an electric circuit that describes a simplified human circulatory system [37], through the definition of a user-defined function written in C language and compiled by Ansys^th^ Fluent^th^. The parameters of the Windkessel model are proximal resistance ($R_{p}$), a distal resistance ($R_{d}$) to implement the vasculare resistances and capacitor vessels (*C*) [38] and the RCR Windwessel equation is given by:

(1) $$P\left(t\right)+R_{d}C\frac{dP(t)}{dt}=\left(R_{p}+R_{d}\right)Q\left(t\right)+R_{p}R_{d}C\frac{dQ(t)}{dt}$$

where *Q* is the flow rate through the vessel (analogue of electric current), and *P* is the patient-specific outlet pressure (analogue of electric voltage). The RCR parameters were estimated iteratively to obtain a numerical solution of Equation (1) matching a pressure waveform corresponding to the patient-specific values of systolic and diastolic pressure.

These parameters are then distributed in the different outlets (Table 1) based on the ratio between the outlet areas [34,39]:

(2) $$R_{p_{i}}=R_{p}\frac{A_{\mathrm{tot}\;}}{A_{i}}$$

(3) $$R_{d_{i}}=R_{d}\frac{A_{\mathrm{tot}\;}}{A_{i}}\;\;\mathrm{for}\;i=BCA,LCCA,LSA,DA$$

(4) $$C_{i}=C\frac{A_{i}}{A_{\mathrm{tot}\;}}$$

Regarding the numerical setup, a time step of 0.004 s was adopted with 50 iterations for each time step. The SIMPLE solution methods were adopted in the solver setup to solve the pressure–velocity coupling and the second-order upwind discretization was performed for the involved equations. A minimum residual convergence of 1 × 10^−5^ has been prescribed. The simulations were performed for three cardiac cycles, each lasting 0.85 s and the results were analyzed for the last cycle, where they achieved stability with variation lower than 0.06% with respect to the previous cycle. In this way, the potential transient effects present at the beginning of the calculation were avoided. For the three configurations of the TA models, the same boundary conditions and simulation setup were applied to ensure that the potential differences in the results derived only from the morphological changes due to the intervention procedures.

Flow rates were extracted from simulation results for all aortic outlets and in particular the differences at LSA level were evaluated for the different configurations as well as the pressure drop between the aortic arch and the LSA extremity at the systolic peak. Since the wall shear stress (WSS) plays crucial role in the arterial disease pathogenesis and progression [40], this parameter was investigated to assess aorta hemodynamics together with the evaluation of the main WSS-based indicators: time averaged wall shear stress (TAWSS), Oscillatory Shear Index (OSI) and Endothelial Cell Activation Potential (ECAP). TAWSS was calculated by integrating WSS magnitude over the cardiac cycle for all configurations [41] as described by the following equation:

(5) $$\mathrm{TAWSS}=\frac{1}{T}\int_{0}^{T}\left|\mathrm{WSS}\left(s,t\right)\right|\cdot dt$$

where *T* is the duration of the cardiac cycle, *s* is the node of the wall mesh, and *t* indicates the time step. Low values of TAWSS (<0.4Pa) have been associated with a region more prone to atherosclerosis [42], since WSS affects the function of the endothelial cells [43]. The OSI indicator describes the changes in the orientation of the WSS vector from the predominant blood flow direction along a cardiac cycle [44] and was calculated according to:

(6) $$\mathrm{OSI}=0.5\left(1-\frac{\left|\int_{0}^{T}\mathrm{WSS}\left(s,t\right)\cdot dt\right|}{\int_{0}^{T}\left|\mathrm{WSS}\left(s,t\right)\right|\cdot dt}\right)$$

The values of the OSI parameter can range between 0 (no oscillation of the WSS vector) and 0.5 (a high oscillating shear stress). High values of OSI (>0.25) have been associated with atherogenesis [45]. The ECAP indicator has been adopted to identify regions characterized by both low TAWSS and high OSI associated to a thrombogenic risk [46] since it is defined as the ratio between OSI and TAWSS:

(7) $$\mathrm{ECAP}=\frac{\mathrm{OSI}}{\mathrm{TAWSS}}$$

## 3. Results

### 3.1. Image Processing and Morphological Analysis

**Figure 3:**
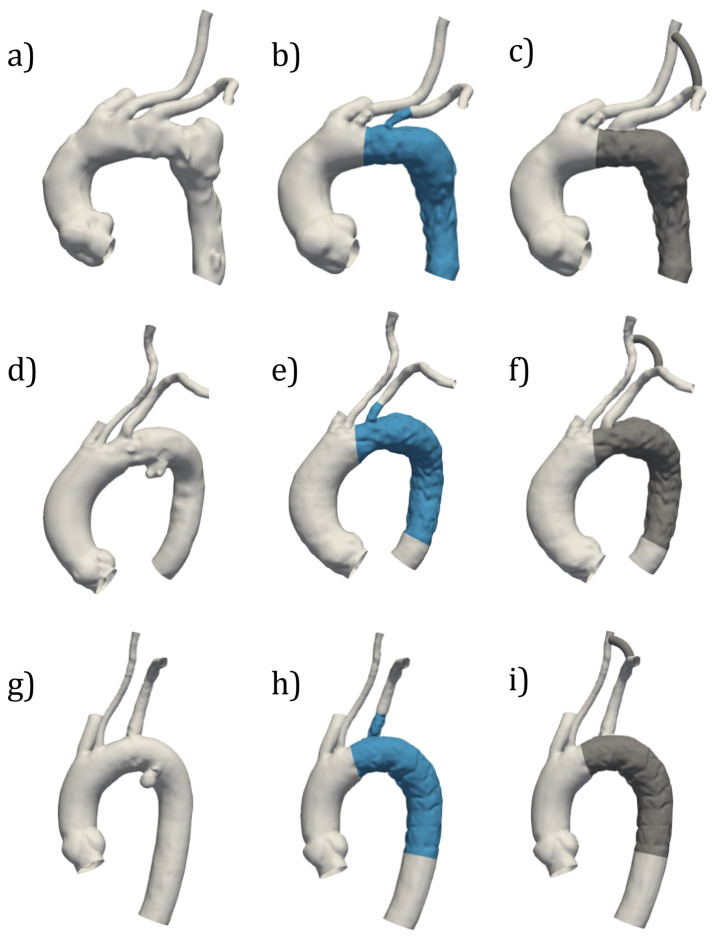
Three-dimensional models of TA for PRE (**a**,**d**,**g**), SBSG (**b**,**e**,**h**) and HYBRID (**c**,**f**,**i**) configurations for CASE 1 (**a**–**c**), CASE 2 (**d**–**f**), and CASE 3 (**g**–**i**).

The reconstruction of PRE and SBSG configurations from segmentation of CT images was feasible for all cases (Figure 3). For the SBSG configurations, it is possible to notice effects of the endograft on the distal aortic arch, LSA, and DA. HYBRID configurations for all cases were obtained by the CAD procedure and are reported in Figure 3c,f,i.

**Figure 4:**
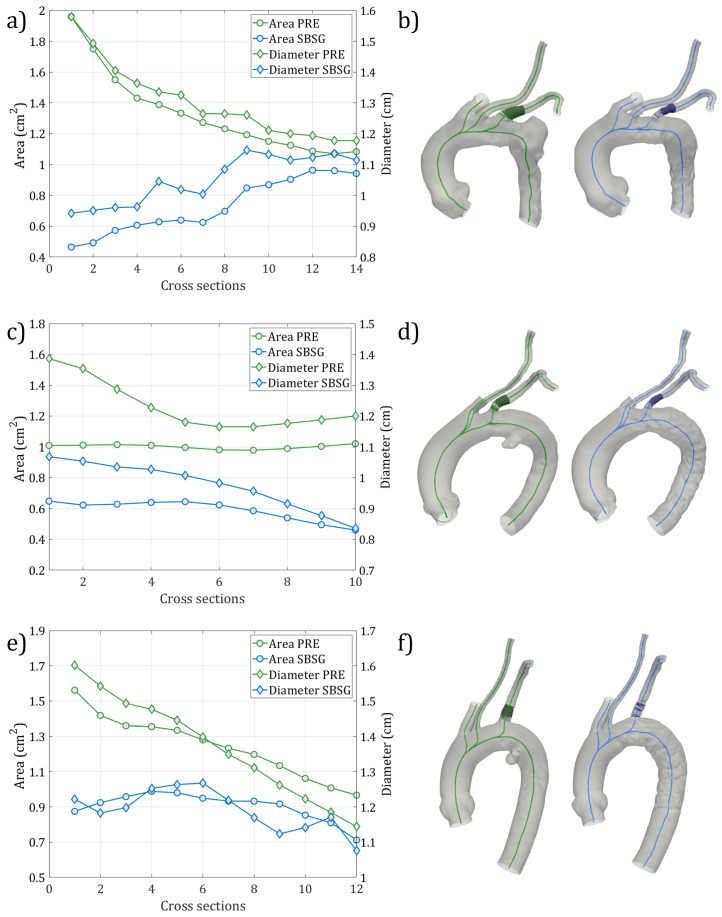
Cross-sectional areas and maximum diameters of the extracted sections for the LSA for PRE (in green) and SBSG (in blue) configurations for CASE 1 (**a**,**b**), CASE 2 (**c**,**d**), and CASE 3 (**e**,**f**).

The cross-sectional maximum diameters and areas of the extracted sections for the LSA for PRE (in green) and SBSG (in blue) geometries for all cases are depicted in Figure 4. The $D_{max}$ differences between PRE and SBSG covered a range from 0.04 cm to 0.64 cm for CASE 1, from 0.17 cm to 0.36 cm for CASE 2, and from 0.01 cm to 0.38 cm for CASE 3. The differences in terms of cross-section areas were found over the ranges of 0.11–1.49 cm^2^, 0.35–0.56 cm^2^, and 0.20–0.69 cm^2^ for CASE 1, CASE 2, and CASE 3, respectively. Table 2 reports the average $D_{max}$, the LSA bifurcation angle θ, and the tortuosity values for all configurations and cases.

### 3.2. Hemodynamics Analysis

**Figure 5:**
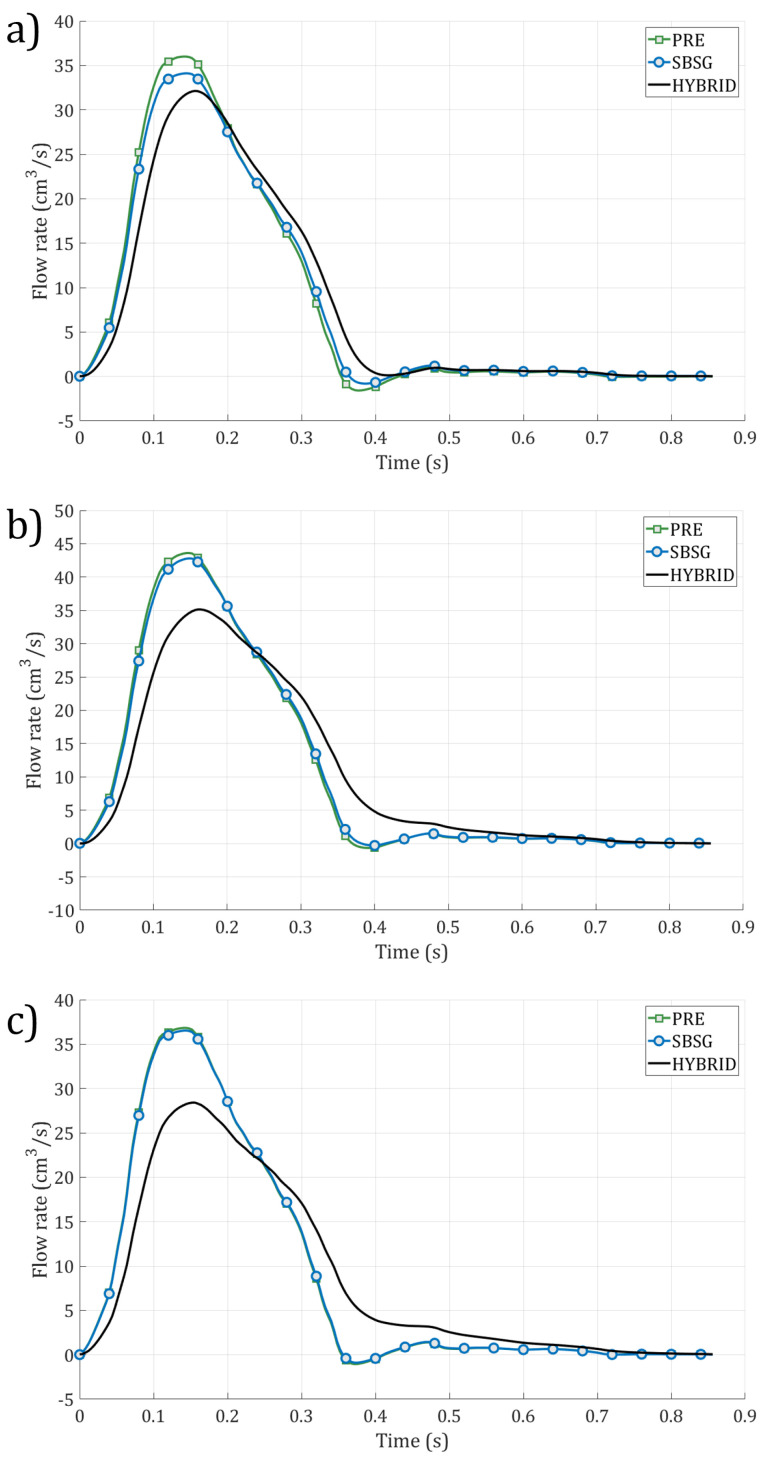
Flow rate at LSA for the three configurations for CASE 1 (**a**), CASE 2 (**b**), and CASE 3 (**c**).

Fluid dynamic results in terms of flow rate at LSA outlet were extracted for each geometry for all cases and are depicted in Figure 5.

**Figure 6:**
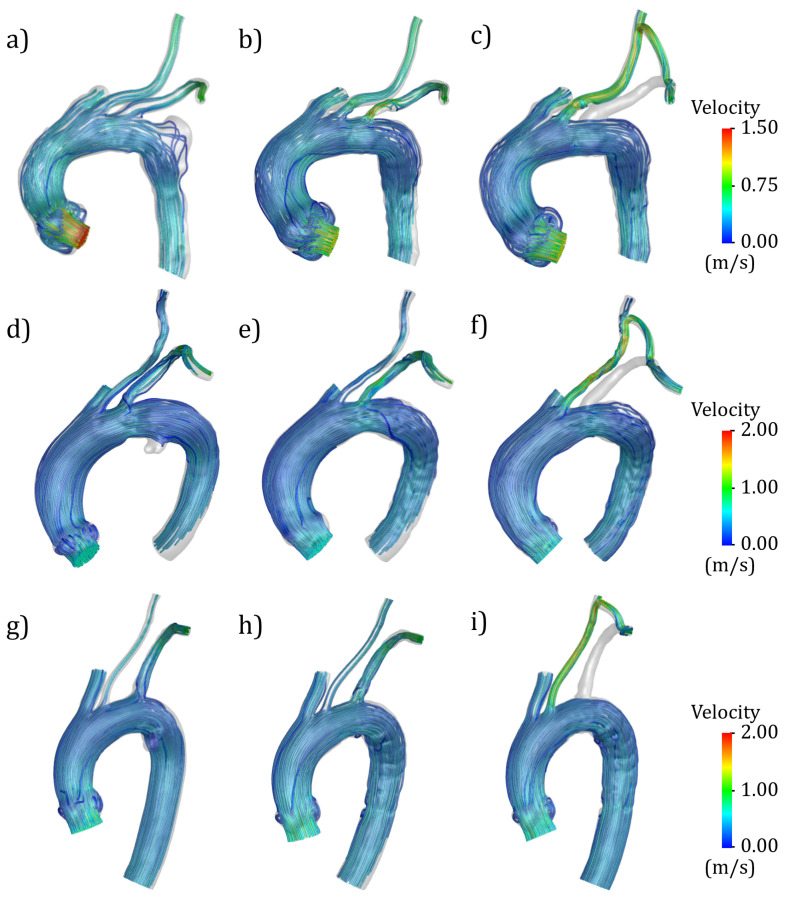
Velocity streamlines at systolic peak for PRE (**a**,**d**,**g**), SBSG (**b**,**e**,**h**), and HYBRID (**c**,**f**,**i**) configurations for CASE 1 (**a**–**c**), CASE 2 (**d**–**f**), and CASE 3 (**g**–**i**).

Velocity streamlines at systolic peak instant are depicted in Figure 6, providing a qualitative assessment of changes in blood flow patterns and velocity values due to the PAU treatment with different strategies. SBSG geometries showed higher velocity values at the LSA level (Figure 6b,e,h), while for the HYBRID, streamlines were not present in the proximal region of LSA and higher velocities were visible along the LCCA lumen and at the beginning of the bypass conduit (Figure 6c,f,i).

The pressure drop values are reported in Table 3 and revealed a minimum for each case in the PRE configuration and a maximum for HYBRID configuration.

**Figure 7:**
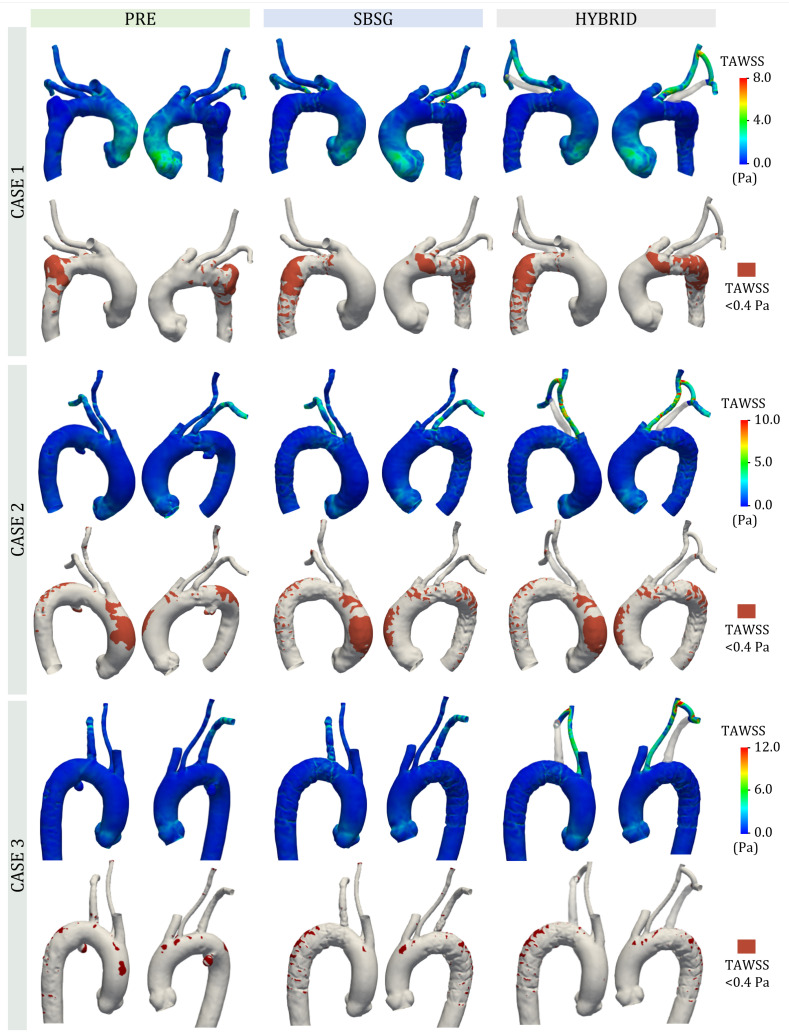
For each case, TAWSS distribution (**top**) and area exposed to low TAWSS values (<0.4 Pa) (**bottom**).

The TAWSS distributions are reported in Figure 7 for all PAU strategies and cases, together with the area exposed to low values of TAWSS (<0.4 Pa). The higher TAWSS values are located in the regions of smaller lumen of supra-aortic branches for SBSG and HYBRID configurations for all cases. A lower TAWSS is located in the PAU region of PRE configurations for all cases. Moreover, regarding CASE 1, low values of TAWSS are present in the proximal aortic arch, at the extrados of distal arch and of the top of the DA and in the grafted DA for the configurations after the TEVAR procedure. CASE 2 showed low TAWSS regions at the extrados of ascending aorta and of the proximal DA. For SGSB and HYBRID configurations of CASE 3, low TAWSS values are present in the grafted DA.

The areas exposed to low TAWSS, normalized to each TA area, for all configurations and cases are reported in Table 4.

**Figure 8:**
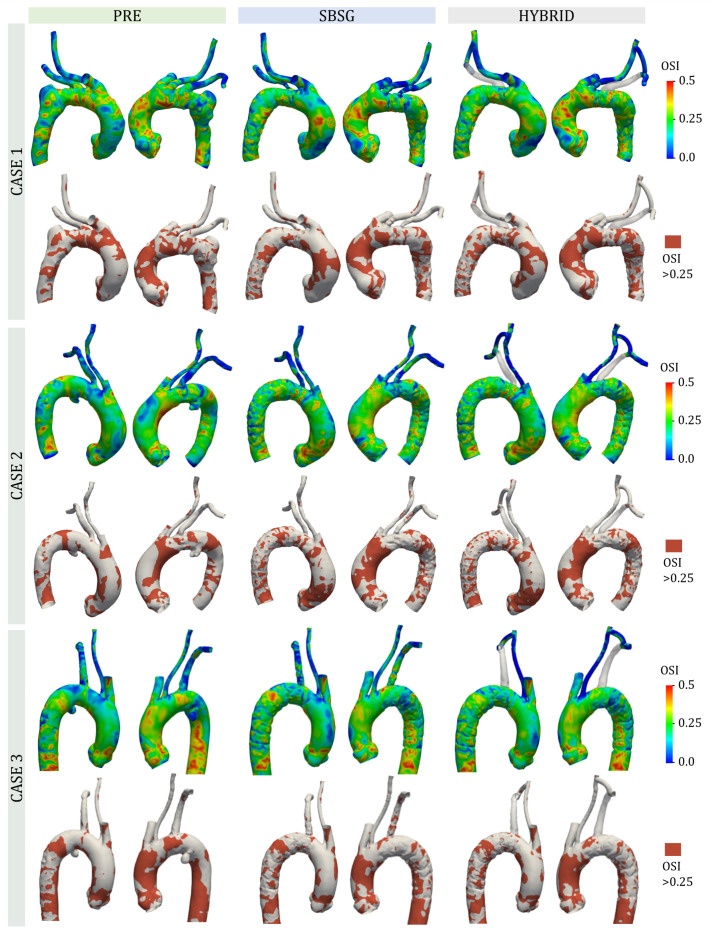
For each case, OSI distribution (**top**) and area exposed to high OSI values (>0.25) (**bottom**).

Figure 8 depicts the effects of TEVAR interventions on the OSI indicator in terms of surface distribution and area exposed to high OSI values (>0.25). CASE 1 showed high OSI values mainly located at the arch and ascending anterior aorta for PRE configuration, while the TAs after intervention revealed high OSI also in the extrados of ascending aorta and lower OSI in the posterior region of the arch. CASE 2 is characterized by high OSI values in the intrados of ascending aorta for PRE configuration, which also extend to anterior ascending aorta in the medial region and to the posterior part of the aortic root for SBSG and HYBRID. CASE 3 showed a distribution of high OSI values quite similar among all configurations with a greater concentration in the anterior ascending aorta, DA, and anterior portion of the arch.

Areas exposed to OSI > 0.25, normalized to each TA area, for all configurations and cases are reported in Table 4.

**Figure 9:**
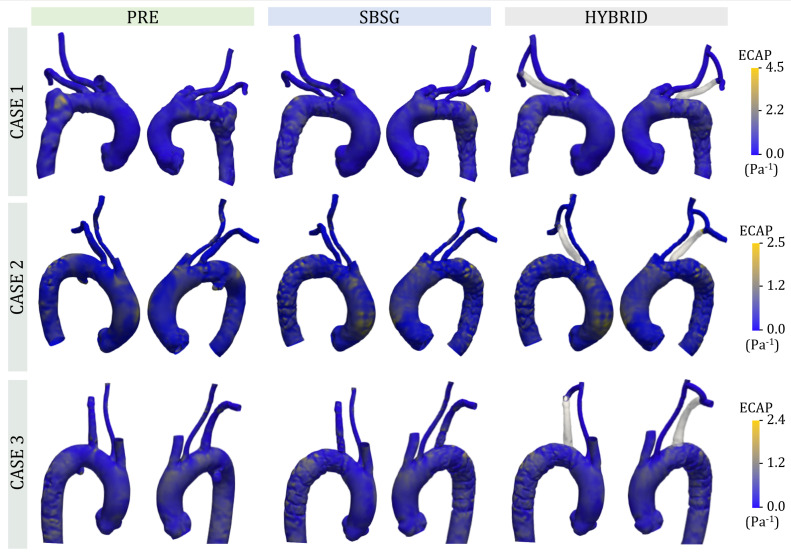
Comparison of ECAP distributions for each case for PRE, SBSG, and HYBRID configurations.

ECAP distributions are depicted in Figure 9 and reveal overall values below 4.5 ${\mathrm{Pa}}^{-1}$. CASE 1 showed high ECAP in the PAU region for PRE configuration and in the anterior arch and DA after the TEVAR procedure. High ECAP was found in PAU and in the extrados and intrados of the central ascending aorta in the PRE configuration for CASE 2, while for the SBSG and HYBRID configurations high ECAP values were also present in the posterior region of the proximal ascending aorta. The higher ECAP values of CASE 3 were localized in the PAU, the anterior part of the distal ascending aorta and the arch, and at the distal DA, while the configurations after TEVAR revealed high ECAP more concentrated in the proximal DA.

## 4. Discussion

This study demonstrates the feasibility of using CFD simulations to analyze hemodynamic changes and vessel morphology after PAU endovascular treatment, revealing that the branched aortic endograft does not significantly affect the blood flow at the LSA, while the hybrid approach with TEVAR and carotid-LSA bypass results in higher pressure drops. PAU is a severe form of acute aortic syndrome that carries a high risk of rupture, particularly in older individuals with comorbidities. Due to the complexities of the condition and the risks associated with open surgery, especially in older patients and when it involves the ascending aorta or the aortic arch, endovascular approaches like TEVAR [47] are increasingly preferred for their minimally invasive nature and lower perioperative risks. If there is a need to protect blood flow in an epiaortic vessel, such as the LSA, TEVAR can be performed using a hybrid approach that includes creating a bypass (e.g., LCCA to LSA) [12] or with a completely endovascular approach using a branched endograft [13,48,49]. Any procedure leads to changes in aorta geometry and consequently in the artery hemodynamics. The assessment of hemodynamic parameters is a crucial factor for the genesis, progression, and prediction of cardiovascular diseases and it is often performed by numerical simulations. The role of numerical simulations in the investigation of cardiovascular hemodynamics and device adoption is indeed widely recognized [50,51,52]. CFD simulations, which implement the hypothesis of rigid walls for the vessels, are widespread in the literature also for the investigation of an endovascular procedure on aorta hemodynamics [53,54,55]. On the other hand, the FSI method can be adopted to study the mutual effects between vessel wall, hemodynamics, and stent graft [56,57]. Nevertheless, it requires definition of the material properties of the artery that are often not available due to the difficulty with respect to accurately estimating them in vivo. Therefore, these properties are often assumed from the literature, introducing a source of uncertainty in the model setup. Despite the limit of CFD in the consideration of vessel motion and distensibility, this approach is more sensitive to high WSS and pressure drop; thus, it can be considered the safest in the hemodynamic parameter estimation when the patient-specific stiffness, motion, and other material properties are not available [58].

In this work, the consequences of innovative adoption of an SBSG, including a component for the LSA, on the LSA flow rate have been studied by performing CFD simulations based on CT images together with a comparison with the effects on blood flow due to the hybrid treatment with endograft and LCCA-LSA bypass. Given the importance of the impact of TA geometry and hemodynamics [15,39,59], we also evaluated the TA morphology to investigate the relations between shape changes of TA, particularly the LSA branch, due to endovascular PAU treatment with the pre-cannuled LSA branch and the fluid dynamics. The TA configurations before the intervention and after the endovascular procedure were obtained directly from patient medical images, while a CAD approach was employed to create a virtual TA configuration after a PAU hybrid treatment involving endograft and LCCA-LSA bypass.

The morphological analysis showed that the average relative reduction of the lumen area was higher for CASE 1 and CASE 2 (41.26% and 41.18%), while CASE 3 revealed the minimum decrease of 26.60%. This aspect may be associated with the different tortuosity and bifurcation angle values for the three cases (Table 2). CASE 3 showed a higher θ and lower tortuosity, turned out in a more vertical vessel compared to the other cases, where LSA resulted in a greater incline toward the aortic arch. Indeed, in these conditions the branch of the endograft is subject to bending in order to stick to the vessel wall leading to a greater lumen reduction.

The evaluation of the flow rate through LSA by analyzing CFD simulation results showed a minimal reduction after the SBSG endovascular procedure compared to PRE configuration equal to 2.38%, 0.63%, and 0.23% for CASE 1, CASE 2, and CASE 3, respectively. Regarding the reconstructed HYBRID configuration, decreases amounting to 3.81%, 7.74%, and 10.97% with respect to the PRE configuration were found for CASE 1, CASE 2, and CASE 3, respectively. These results highlight the conservation of blood flow in LSA with the usage of SBSG compared to the hybrid strategy.

The highest flow rate decrease was observed in CASE 1, which also showed the highest reduction in LSA lumen size (Figure 4) at the vessel origin. The effects of different types of PAU treatment on the TA hemodynamics were also highlighted by the velocity streamlines (Figure 6), confirming the impact of geometric factors on blood flow dynamics [60,61]. Regarding the pressure drop between the aortic arch and the LSA outlet, the SBSG geometries exhibited higher values than the respective PRE, whereas the highest values were reached in the HYBRID configurations (Table 3), suggesting that the TEVAR procedure causes an increase in the pressure drop at the LSA. The major increment in the SBSG configurations was reached in CASE 1 (about double the pressure drop before the intervention), which is related to the more pronounced reduction in the LSA diameter. Nevertheless, the absolute values of the pressure drop after the intervention for the SBSG configurations still remained not clinically relevant.

The role of WSS-based indicators in the hemodynamics assessment is well recognized in the literature since they affect endothelial proliferation; in particular, low values of TAWSS indicator and high values of OSI have been associated with an increase in atherogenesis risk [44,62]. Both spatial distributions of TAWSS and OSI and quantitative evaluation of the area exposed to low TAWSS (<0.4 Pa) and high OSI (>0.25) values were performed in this study (Figure 7 and Figure 8 and Table 4).

Low TAWSS values were found in the PAU regions due to the lower blood flow velocity in the aneurysmatic sacs. The presence of low TAWSS also in the ascending aorta for CASE 2 is caused by the larger lumen size of this patient with respect to the other two cases according to previous literature results [60]. Areas exposed to low TAWSS, normalized to each TA area, were found similar for the corresponding configurations of CASE 1 and CASE 2, while the lower area for CASE 3 is due to the lower vessel size. The low TAWSS area of postoperative configurations increased with respect to the PRE condition for CASE 1 (36.4% for SBSG and 52.5% for HYBRID) and CASE 2 (36.2% for SBSG and 31.5% for HYBRID). This increase could make the involved regions of TA more prone to atherosclerosis after the LSA revascularization, as reported in previous studies which evaluate the effect of the TEVAR procedure on the TA hemodynamics [27,63]. The absence of differences pre and after intervention for CASE 3 is potentially due to its lower variation in LSA lumen area and consequently in flow pattern redistribution.

The area exposed to high OSI turned out to be quite similar for all cases with a very low variation between PRE configuration and configurations after TEVAR for CASE 1 and CASE 3 and an increase for CASE 2 of 17.7% and 20.3% for SBSG and HYBRID configurations, respectively.

The ECAP indicator was adopted for the evaluation of the combined effects of TAWSS and OSI, allowing the localization of regions affected by both low TAWSS and high OSI. Previous research reports that high values of ECAP (>1.4$\;{\mathrm{Pa}}^{-1}$) identified regions more prone to endothelial cell deposition and values $>5\;{\mathrm{Pa}}^{-1}$ in the aneurysmatic region were found to be associated with thrombus formation [46]. Our results showed an agreement with the literature [64,65], since the highest ECAP values, closer to 5 ${\mathrm{Pa}}^{-1}$, were found in the PAU in the PRE configurations, while other regions were characterized by lower ECAP and no significant differences were found between PRE and post-intervention configurations (Figure 9).

Further improvements in this work may concern the morphological and fluid dynamic analysis of a greater number of cases covering a wide spectrum of aortic arch and supra-aortic vessel geometries as well as different post-intervention configurations. It is well known that placement, size, orientation, and morphology of cardiovascular endograft and shunts can induce different hemodynamic alterations in the anatomical districts [66,67,68]. In the near future, a wider adoption of photon-counting computed tomography might significantly impact pre-procedural evaluations [69,70] thanks to its high resolution and ability to detect stent struts [71]. Although in this work the creation of a HYBRID configuration was carried out following the surgeon’s criteria, particularly in terms of size and placing, several conduit conformations could be investigated by also exploring different bypass diameters, lengths, and anastomosis angles with LCCA and LSA through CFD simulations [72]. In this context, a statistical shape model approach [73] could represent a powerful instrument to obtain a set of TA with supra-aortic vessel geometries and LCCA-LSA bypass configurations. Previous studies [74,75,76] have highlighted the clinical benefits of the SBSG procedure for treating acute aortic pathologies. In our study, we employed image-based CFD to perform a comparative analysis. Future work could include post-intervention, patient-specific simulations, incorporating echocardiographic assessment of flow rates in the supra-aortic branches alongside aortic geometry reconstruction. This approach would provide a more comprehensive evaluation of the procedure’s efficacy from a clinical perspective.

## 5. Conclusions

The morphological analysis offers deeper insights into the effects of endografts on the TA, establishing it as a valuable tool for personalized medicine. CFD simulations based on CT images suggest that a single-branched endograft may be more effective at preserving LSA flow compared to the hybrid TEVAR LCCA-LSA bypass procedure, together with a lesser increment of pressure drop. The performance of PAU treatment appears to improve thanks to fully endovascular single-branch stent graft techniques, also considering the potential advantages due to the less invasive nature of the method. However, these findings are based on computational modeling applied to a specific anatomic setting and require further validation to confirm their reliability in broader real-world settings. Additionally, future studies incorporating clinical outcomes and long-term follow-up are essential to assess the prognostic impact of these techniques.

## Figures and Tables

**Table 1:** RCR parameters: distal resistance ($R_{d}$), proximal resistance ($R_{p}$), and compliance (*C*) for all outlets for the three cases. Resistances are expressed in $\mathrm{kg}\;m^{-4}\;s^{-1}$ and compliance terms in ${\mathrm{kg}}^{-1}\;m^{4}\;s^{2}$.

	CASE 1			CASE 2			CASE 3		
	$R_{d}$	$R_{p}$	C	$R_{d}$	$R_{p}$	C	$R_{d}$	$R_{p}$	C
BCA	$5.62\times 10^{8}$	$5.08\times 10^{7}$	$2.42\times 10^{-9}$	$8.81\times 10^{8}$	$7.98\times 10^{7}$	$1.54\times 10^{-9}$	$8.87\times 10^{8}$	$8.03\times 10^{7}$	$2.06\times 10^{-9}$
LCCA	$1.44\times 10^{9}$	$1.31\times 10^{8}$	$9.42\times 10^{-10}$	$3.39\times 10^{9}$	$3.07\times 10^{8}$	$4.02\times 10^{-10}$	$3.61\times 10^{9}$	$3.27\times 10^{8}$	$3.77\times 10^{-10}$
LSA	$1.37\times 10^{9}$	$1.24\times 10^{8}$	$9.96\times 10^{-10}$	$1.06\times 10^{9}$	$9.62\times 10^{7}$	$1.28\times 10^{-9}$	$1.29\times 10^{9}$	$1.16\times 10^{8}$	$1.06\times 10^{-9}$
DA	$2.51\times 10^{8}$	$2.28\times 10^{7}$	$5.41\times 10^{-9}$	$2.08\times 10^{8}$	$1.88\times 10^{7}$	$6.55\times 10^{-9}$	$2.00\times 10^{8}$	$1.81\times 10^{7}$	$6.80\times 10^{-9}$

**Table 2:** Morphological parameters for all configurations for the three considered cases.

	CASE 1		CASE 2		CASE 3	
	**PRE**	**SBSG**	**PRE**	**SBSG**	**PRE**	**SBSG**
Average $D_{max}$ (cm)	1.30	1.05	1.23	0.98	1.37	1.20
Average Area (${\mathrm{cm}}^{2}$)	1.33	0.73	1.00	0.59	1.24	0.90
*θ*°	43.26	44.48	58.69	57.43	86.92	78.17
Tortuosity	0.91	0.99	0.61	0.66	0.29	0.27

**Table 3:** Pressure drop in mmHg between aortic arch and LSA extremity at systolic peak for all configurations for each case.

	CASE 1	CASE 2	CASE 3
PRE	3.53	8.18	5.00
SBSG	6.95	9.69	5.53
HYBRID	15.21	23.51	21.14

**Table 4:** Area exposed to TAWSS <0.4Pa ($Area_{TAWSS}$) and area exposed to OSI >0.25 ($Area_{OSI}$) normalized with respect to area of each TA configuration and expressed in %.

	CASE 1			CASE 2			CASE 3		
	**PRE**	**SBSG**	**HYBRID**	**PRE**	**SBSG**	**HYBRID**	**PRE**	**SBSG**	**HYBRID**
$Area_{TAWSS}$	11.8	16.1	18.0	12.7	17.3	16.7	4.3	4.2	4.3
$Area_{OSI}$	33.1	31.0	33.4	30.5	35.9	36.7	37.4	34.6	35.6

## Data Availability

The data presented in this study are available on reasonable request from the corresponding author. The data are not publicly available due to privacy restrictions.
